# Oral appliance therapy and hypoglossal nerve stimulation as non-positive airway pressure treatment alternatives for obstructive sleep apnea: a narrative expert review

**DOI:** 10.1093/sleepadvances/zpae035

**Published:** 2024-06-14

**Authors:** Sairam Parthasarathy, Najib T Ayas, Richard Bogan, Dennis Hwang, Clete Kushida, Jonathan S Lown, Joseph M Ojile, Imran Patel, Bharati Prasad, David M Rapoport, Patrick Strollo, Oliver M Vanderveken, John Viviano

**Affiliations:** Department of Medicine, University of Arizona Health Sciences Center for Sleep and Circadian Sciences, University of Arizona, Tucson, AZ, USA; Department of Medicine, University of British Columbia, Vancouver, BC, Canada; Department of Psychaiatry and Behavioral Sciences, Medical University of South Carolina, Charleston, SC, USA; Division of Sleep Medicine, Kaiser Permanente Southern California, Fontana, CA, USA; Division of Sleep Medicine, Department of Psychiatry and Behavioral Sciences, Stanford University, Stanford, CA, USA; Delta Sleep Center of Long Island, Commack, NY, USA; Clayton Sleep Institute, St. Louis, MO, USA; Department of Medicine, University of Arizona Health Sciences Center for Sleep and Circadian Sciences, University of Arizona, Tucson, AZ, USA; Dental Sleep Service Line, Banner University Medical Center - Tucson, Tucson, AZ, USA; Department of Medicine, University of Illinois at Chicago and Jesse Brown VA Medical Center, Chicago, IL, USA; Mount Sinai Health System Integrative Sleep Center, Icahn School of Medicine at Mount Sinai, New York, NY, USA; Division of Pulmonary, Allergy, and Critical Care Medicine University of Pittsburgh School of Medicine, Pittsburgh, PA, USA; Department of ENT-HNS, Antwerp University Hospital, Edegem, Belgium and Translational Neurosciences, Faculty of Medicine and Health Sciences, University of Antwerp, Antwerp, Belgium; Sleep Disorders Dentistry Research and Learning Center, Mississauga, ON, Canada

**Keywords:** sleep apnea, obstructive sleep apnea, positive airway pressure, oral appliance, dental appliance, hypoglossal nerve stimulation, sleep, implementation science, dental, adherence

## Abstract

This perspective on alternatives to positive airway pressure (PAP) therapy for the treatment of obstructive sleep apnea (OSA) summarizes the proceedings of a focus group that was conducted by the Sleep Research Society Foundation. This perspective is from a multidisciplinary panel of experts from sleep medicine, dental sleep medicine, and otolaryngology that aims to identify the current role of oral appliance therapy and hypoglossal nerve stimulation for the treatment of OSA with emphasis on the US practice arena. A secondary aim is to identify—from an implementation science standpoint—the various barriers and facilitators for adoption of non-PAP treatment that includes access to care, multidisciplinary expertise, reimbursement, regulatory aspects, current treatment guidelines, health policies, and other factors related to the delivery of care. The panel has contextualized the review with recent events—such as a large-scale PAP device recall compounded by supply chain woes of the pandemic—and emerging science in the field of OSA and offers solutions for multidisciplinary approaches while identifying knowledge gaps and future research opportunities.

Obstructive sleep apnea (OSA) affects approximately 12% of the US population and is characterized by repetitive collapse of the upper airway during sleep [1]. OSA is independently associated with systemic hypertension [2–4], cardiovascular disease [5–7], stroke [8], reduced health-related quality of life (HR-QOL) [9], increased mortality [8, 10, 11], and motor vehicle accidents due to sleepiness [10, 12–14]. Positive airway pressure (PAP) therapy is commonly used to treat OSA, and clinical practice guidelines recommend PAP therapy strongly for the treatment of OSA. However, non-PAP alternatives such as oral appliance therapy (OAT) and Hypoglossal Nerve Stimulation (HNS) are available but not widely adopted. A recent report from a nationwide survey of 2289 patients with OSA suggested that OAT was used in only 6% of patients and HNS in 0.3% [15]. This narrative review summarizes the proceedings of a focus group that was conducted by the Sleep Research Society Foundation (SRSF) that was attended by a multidisciplinary panel of experts from sleep medicine, otolaryngology, and dental sleep medicine with the primary objective of identifying the role of dental devices and HNS for the treatment of OSA with emphasis on the US practice arena. The secondary objectives were to identify and address the various barriers and facilitators for the adoption of non-PAP treatment approaches that include access to care; the need for multidisciplinary expertise; reimbursement and regulatory issues; current practice guidelines; health policies and other factors related to delivery of care. This document is not an exhaustive review of this topical area and should not be used as a practice standard. The expert panel was selected by seeking nominations from Key Opinion Leaders who are experts in the field who had published in the area of OAT, HNS, and PAP therapy for OSA. We also included practitioners who are actively providing care using OAT, HNS, and PAP therapy so as to better understand their expert opinion of “real-world” barriers and facilitators for non-PAP therapy.

In 2015, the American Academy of Sleep Medicine (AASM) and the American Academy of Dental Sleep Medicine (AADSM) Board of Directors recommended that sleep physicians consider prescription of OAT, rather than no treatment, for adult patients with OSA who are intolerant of PAP therapy or prefer alternate therapy [16]. Moreover, an AASM task force of experts in sleep medicine, otolaryngology, and bariatric surgery made a strong recommendation for sleep medicine providers to discuss referral to a sleep surgeon when caring for adults with OSA and body mass index (BMI) < 40 kg/m2 who are intolerant or unaccepting of PAP following a patient-oriented discussion of alternative treatment options [17]. A “Strong” recommendation, as opposed to a “conditional” recommendation, is one that clinicians should follow for almost all patients. The recommendation did not preclude consideration of other non-PAP alternatives such as OAT, positional therapy, or lifestyle changes. In individuals with BMI > 35 Kg/m2, considerations for bariatric surgery as a treatment option can be discussed and referral to bariatric surgeons may be considered [17]. Considering such recommendations for referrals to surgeons or dentists, better definition of the role of OAT and HNS in the treatment of OSA while addressing “real world” barriers and facilitators for adoption of non-PAP treatment approaches was warranted. Our focus group of experts shared important perspectives and identified knowledge gaps to enable a better understanding of the environment of care and contextual factors that may impact implementation of non-PAP alternatives [18]. This white paper addresses various thematic domains that includes (1) identifying general goals of therapy for patients with OSA to contextualize the discussion; (2) identifying the extent and nature of PAP intolerance or PAP failure in patients with OSA; (3) discussing when non-PAP treatment should be considered as first-line treatment; (4) addressing barriers and facilitators for the adoption and implementation of non-PAP treatments for OSA within a multidisciplinary care model in “real-world” practice settings and contrasting such a model to clinical trial settings; (5) acknowledging limitations of this initiative; and (6) identifying future directions (see Appendix for the probes that were used in these thematic domains). A recording of the rich discussions that served as the nidus for this white paper is available [19].

## Goals of Therapy

Before defining treatment failure or intolerance of PAP therapy for OSA, it would behoove us to define the goals of therapy for OSA. The goals of OSA treatment would be to accomplish the following: (1) Patient-reported outcomes that include patient satisfaction (improvements in daytime sleepiness, vigilance, sleep interruptions, loud snoring, and witnessed apnea); (2) Minimize respiratory events of OSA that are measurable by sleep study or PAP device output (hypoxia, apnea–hypopnea index, sleep interruptions); (3) Achieve adequate PAP adherence metrics to realize the full benefits of PAP efficacy (i.e. effectiveness); and (4) Improvements in associated comorbidities (e.g. blood pressure and cognitive impairments) whenever feasible. Notably, the goals of care need to be discussed and decided with the patient by the sleep medicine provider through combined decision-making [20]. These goals of therapy are multi-dimensional and complex, and each goal poses a host of questions regarding the best approach to measure and an in-depth discussion is better suited in the following section regarding treatment failure.

## Definition of Treatment Failure

Various practices have their own approach to initiating treatment. Whilst many practices commence with PAP therapy because they have existing working relationships with Durable Medical Equipment (DME; or homecare) companies that can easily initiate the PAP therapy in the patient’s home or at a DME “walk-in” facility, other practices have well-established relationships with dental sleep medicine or surgical providers and are able to initiate OAT or surgical approaches for managing OSA. Most sleep medicine practices have staff or programs that monitor and promote PAP adherence, but such an endeavor involves resources (personnel and facility charges) and additional clinic visits for troubleshooting PAP intolerance. Staffing of such adherence promotion endeavors include, but are not limited to clinical psychologists, trained respiratory therapists, experienced nurses, text platforms from PAP manufacturers, patient group therapy, and individualized peer support approaches aimed at promoting adherence [21–33]. However, the time period after which PAP adherence promotion is considered to have failed appears to be highly variable. There are various factors that need to be considered when PAP treatment failure occurs, but remarkably the field does not have a consensus-based definition or guidance for adjudicating PAP treatment failure. Some of the suggested definitions for PAP treatment failure are listed in Table 1.

**Table 1.** Suggested Definitions for PAP Treatment Failure

Patient-centered	
	Patient declines to initiate PAP therapy
	Patient discontinues PAP therapy
	Lack of improvement in primary presenting sleep-related complaints despite adequate treatment adherence
	Significant side-effects (common or rare) that prohibit continuation (e.g. severe epistaxis, severe sinus infections, recurrent pneumothorax, etc.)
	Intolerance of mask, pressure, or entire set-up despite attempts at correcting these issues over time (2 weeks to 90 days)^*^
Provider-assessed	
	Failure to normalize AHI and/or oxygenation due to PAP emergent central apneas^‡^
	Failure to improve sleep continuity and/or daytime sleepiness despite adequate PAP adherence ^@^
	Failure of alternate PAP modalities (bilevel PAP, adaptive servo-ventilation, etc.) to normalize AHI and/or oxygenation.^†^
Payor-based	
	Failure to reach adherence threshold as required by payor’s policy^#^
	Failure of patient to report symptom improvement

^*^Duration of PAP trial may be determined through combined decision-making or predicated by the practice setting and can even extend to 1-year provided the patient is able to maintain access to therapy through self-pay or payor; ^@^ May prompt evaluation for other coexisting sleep disorders or medical conditions including residual sleepiness of OSA and not necessarily consider discontinuing PAP treatment;

^†^Alternate PAP modalities were attempted following laboratory-based polysomnography and PAP device download as per AASM guidelines [34].

^‡^Measured by device downloads or sleep study;

^#^Most payors adopt the Medicare threshold of defining PAP adherence as > 4 hours per day for 5 days a week in a 30 continuous day time period within a 90-day time window. AHI, apnea–hypopnea index; PAP, positive airway pressure.

The most recent practice guideline for the treatment of OSA with PAP therapy [34] addresses combined decision-making as an approach to consider non-PAP alternatives for treatment of OSA, but the AASM Taskforce admittedly did not (1) compare PAP against other treatment options (e.g. OAT, surgical therapy); nor (2) address the metrics for PAP treatment failure that would warrant non-PAP alternative therapies. However, there were recommendations for when to consider PAP therapy, the type of PAP therapy, and the methods and approaches aimed at promoting PAP adherence.

It is uncertain as to how many practices routinely administer questionnaires aimed at measuring patient-reported outcomes (PROs) and use them as the basis for evaluating treatment success or failure. There is a plethora of well-validated PROs that can reliably measure self-reported complaints of daytime sleepiness, nighttime sleep quality, and sleep-related daytime impairments which could become the basis for adjudicating treatment failure [35–39]. There is scientific evidence for PAP therapy being able to improve health-related quality of life in patients with mild OSA in contrast to lack of supporting data for reducing cardiovascular disease or all-cause mortality [39, 40]. Moreover, Clinical Global Impression scale (-Severity; CGI-S) can enable measurement of a clinician’s assessment of the disease impact on patient’s global functioning and the changes in CGI can be used to denote treatment response [41]. However, adoption of such rigorous approaches to justify treatment decisions in day-to-day practice settings is generally not instituted. Conceivably, the development and implementation of Clinical Decision Support Systems may address barriers in routine practice, but the development and implementation of such Clinical Decision Support Systems are in their infancy in the field of sleep medicine [42, 43].

Adequate PAP adherence metrics need to be accomplished for patients to realize the full treatment benefits of PAP efficacy (i.e. effectiveness) and may vary by target outcome such as improving sleepiness or cardiovascular outcomes (e.g. hypertension). In general, most practices rigorously assess PAP adherence and through combined decision-making with patients and caregivers decide on whether to abandon PAP therapy and pursue OAT. Many factors including patient preference, severity of OSA, presence of orofacial abnormalities, dentition, history of temporomandibular joint problems, access to care, payor coverage, and other criteria may decide the nature and threshold for such decision-making. These factors are elucidated in a later section.

The extent of nonadherence to PAP therapy seems to vary quite widely. Clearly, the magnitude of PAP nonadherence would have major implications on the extent to which the sleep medicine field would need to embrace non-PAP alternatives. There are two aspects of how poor PAP adherence would inform non-PAP alternatives. First, PAP treatment failure would warrant the deployment of non-PAP alternatives. Second, a personalized treatment approach for when to consider OAT as the first line of therapy—in lieu of a trial of PAP therapy—in individuals who are more likely to succeed with OAT should be defined.

Estimating the extent of PAP adherence is a worthwhile pursuit in order to plan the scope of implementation of a multidisciplinary care model. Estimates of PAP adherence based on clinical trials can both over- and under-estimate the actual PAP adherence rates in a clinic-based population. Conceivably, the low PAP adherence noted in clinical trials could have been caused by the exclusion of participants with excessive daytime sleepiness for ethical reasons [44]. Specifically, the participants in the Sleep Apnea Cardiovascular Endpoints (SAVE) trial—which excluded sleepy phenotypes of OSA—manifested low PAP adherence (mean of 3.3 hours/day over 12 months) which is significantly lower than the desired 4 hours/night. Considering that non-sleepy phenotypes are generally less adherent to PAP therapy, the prevalence of PAP adherence in the SAVE trial cannot be extrapolated to a clinic-based population [45–47]. Alternatively, individuals participating in clinical trials are generally more motivated and possible volunteer bias may tend to be associated with greater PAP adherence [48]. For this reason, real-world evidence is needed to plan the scope of implementation of a multidisciplinary care model and conceivably a different threshold than the arbitrary 4-hour threshold may need to be considered based on the resolution of sleepiness and other patient-reported outcomes.

The origins of the 4-hour threshold were arbitrary. However, subsequently, there have been observational studies, meta-analyses, and trials with a priori stated sensitivity analyses that have used a 4-hour threshold to suggest that cardiovascular comorbidities such as cerebrovascular events or even cardiovascular mortality may be less in the PAP treatment group when such a threshold amount of time is accomplished [46, 49, 50]. But the confounding influence of nonadherence to concomitant cardiovascular medications (antihypertensives, cholesterol-lowering agents, etc.) may potentially explain such observations as being attributable to an individual’s trait rather than a treatment effect [51]. Real-world analyses of databases of PAP-derived adherence data indicate a wide range of PAP adherence (47% to 75%) [52–56]. Regardless, based on both clinical trials and real-world studies, the conclusion that nonadherence is a major barrier to PAP treatment is inescapable. In some individuals, such nonadherence may have been further compounded by both the Severe Acute Respiratory Syndrome Coronavirus type 2 (SARS-CoV-2) pandemic, supply chain issues due to the pandemic, and a major PAP device recall [57–59]. Not many practices were well poised for such a circumstance and ideally sleep medicine practices need to integrate all available therapies for OSA—PAP and non-PAP approaches—and not remain “*PAP-centric*.”

In select patients with OSA, combination therapy with OAT and CPAP can be effective in normalizing the apnea–hypopnea index and reducing daytime sleepiness [60, 61]. However, the prior studies have small sample sizes therefore future studies need to explore candidates for such an approach and generalizability of these findings.

## Non-PAP Therapy Related Side-Effects

Combined decision-making with patients needs to take into consideration the risk of serious or long-term side effects of non-PAP therapies. This is especially the case considering that PAP therapies are in general free of long-term serious side-effects. The invasive nature of the HNS therapy can be associated with surgical and conscious sedation-related complications. Although HNS has a low rate of serious adverse effects, they are certainly serious compared to PAP therapy and include hypoglossal nerve injury, platysma muscle weakness and marginal mandibular nerve injury, pneumothorax, hematoma, and infection warranting device removal [62, 63]. Adverse effects are usually minor and infrequently require discontinuation of the oral appliance. Occlusal changes are the major long-term adverse side-effect of the OAT and rarely may interfere with eating and require readjustments or orthodontic treatments [64, 65].

## Non-PAP Alternatives as First-Line Therapy

Is PAP therapy the first-line therapy? Should non-PAP alternatives be offered as first-line therapies in selected patients? In 2015, the AASM and AADSM Board of Directors recommended that sleep physicians consider prescription of OAT, rather than no treatment, for adult patients with OSA who are intolerant of PAP therapy or *prefer* alternate therapy [16]. This clinical practice guideline was based upon a task force assessment of the literature back in 2015, but widespread adoption of such a standard in the real world has not been likely realized. The reasoning for such an implementation gap is based on the recent data that suggests that although ~37% of patients with OSA were not adherent to their PAP devices, only 6% of individuals were receiving OAT therapy [15].

In general, sleep centers need to offer a variety of treatment options as part of patient-centered care [66, 67]. Treatment options should be provided to patients and patient preference should be ascertained and combined decision-making undertaken [20, 66]. In contrast to OAT, HNS is generally not used as first-line therapy due to its invasive nature and expense. However, the expert panel believed that there is a small subset of patient who may benefit from HNS therapy as a first-line treatment. However, such an opinion is at odds with current Medicare coverage policies and AASM practice parameters [17, 68]. The 2021 AASM clinical practice guidelines on surgical management of OSA make a strong recommendation for clinicians to make a surgical referral for adult patients with OSA and BMI < 40 kg/m2 and PAP intolerance or PAP nonacceptance [17]. Nevertheless, selected patients who are likely responders and do not want to lose time and resources in the process of trying PAP therapy before resorting to HNS therapy may be referred to HNS implantation. For example, active-duty military personnel and pilots with an active license may be candidates for HNS rather than PAP therapy. Nevertheless, the cost and reimbursement issues are major barriers for both OAT and HNS approaches.

Anecdotal observations by the experts that they observed in their practices indicated greater adoption of OAT in the fall of 2021 after the Philips PAP device recall accentuated the preexisting pandemic-related supply chain issues that were hampering the manufacturing of PAP devices. However, data to support such observations are lacking. During the time of the recall and the pandemic, there were significant delays (many weeks) in obtaining PAP devices, and seemingly OAT and HNS therapies gained a greater market share. During this time, the adoption of non-PAP alternatives increased to address the needs of patients with a new diagnosis of OSA or those who had stopped using their recalled PAP device. In many ways, the barriers to PAP therapy became a facilitator for non-PAP alternative therapies. While the COVID pandemic and the PAP device recall were hugely unfortunate events, an accidental “natural experiment” should not go without scrutiny and introspection. This is indeed the right juncture for us to examine the barriers and facilitators for adoption and implementation of non-PAP therapies into a multidisciplinary care model to bring about patient-centric care to our patients.

For OAT to be proposed as first-line treatment for adult patients with OSA, we need to first assess the many comparative effectiveness studies of OAT versus PAP therapy [69–71]. In general, PAP therapy was more efficacious at improving indexes of OSA (such as the apnea–hypopnea index and oxygenation), but OAT was associated with greater treatment adherence. These meta-analyses did not reveal any consistent differences in health-related quality of life or subjective sleepiness between PAP and OAT [69–71]. Most of these studies involved patients with mild-to-moderate severity of OSA in the absence of significant cardiopulmonary disease or orofacial abnormalities that prevented the successful institution of OAT. Many observational studies of both OAT and PAP therapy suggest cardiovascular benefits and adequate long-term adherence in ~50% of the patients exposed to such treatments, however, systematic reviews and meta-analyses of RCTs indicate that we need more comparative effectiveness research with sufficient sample size [49, 52, 69, 72, 73]. Recent advances and approaches to measuring adherence to OAT and calculating the effectiveness of therapy (OAT o PAP) as a function of efficacy and adherence (i.e. effectiveness) allow for meaningful comparisons between PAP and OAT with the understanding that although PAP therapy may be more efficacious than OAT, the greater OAT adherence may lead to comparable effectiveness [74, 75]. Moreover, we need to have sufficient sample size to discern the heterogeneity of treatment effects in various subgroups depending on OSA severity, body habitus, and demographics. In sum, there is scientific equipoise for treating patients with OSA of mild-to-moderate severity with OAT versus PAP therapy in the absence of severe cardiopulmonary disease.

HNS therapy has been shown by many studies to be superior to other upper airway surgeries, but a head-to-head comparative effectiveness trial against PAP therapy is lacking [76]. Moreover, concerns regarding the healthcare expenditure towards PAP therapy in a patient with OSA who could potentially fail PAP therapy but also potentially qualify for first-line HNS therapy cannot be ignored. Model-based estimation of cost-effectiveness in patients who have tried and have not responded to CPAP has found HNS therapy to indeed be cost-effective [77]. Adding more complexity to this situation is the fact that the literature evaluating cardiovascular outcomes following HNS therapy is scarce [78]. Also, there is the matter of patient preference when patients may express that they would prefer to attempt HNS as first-line therapy. It remains that the only avenue for changes in this area requires rigorous comparative effectiveness research of PAP and non-PAP alternatives (e.g. HNS therapy) that would then inform coverage policies, practice guidelines, and routine care delivery for patients with OSA.

## Multidisciplinary Care Model

**Figure 1.**
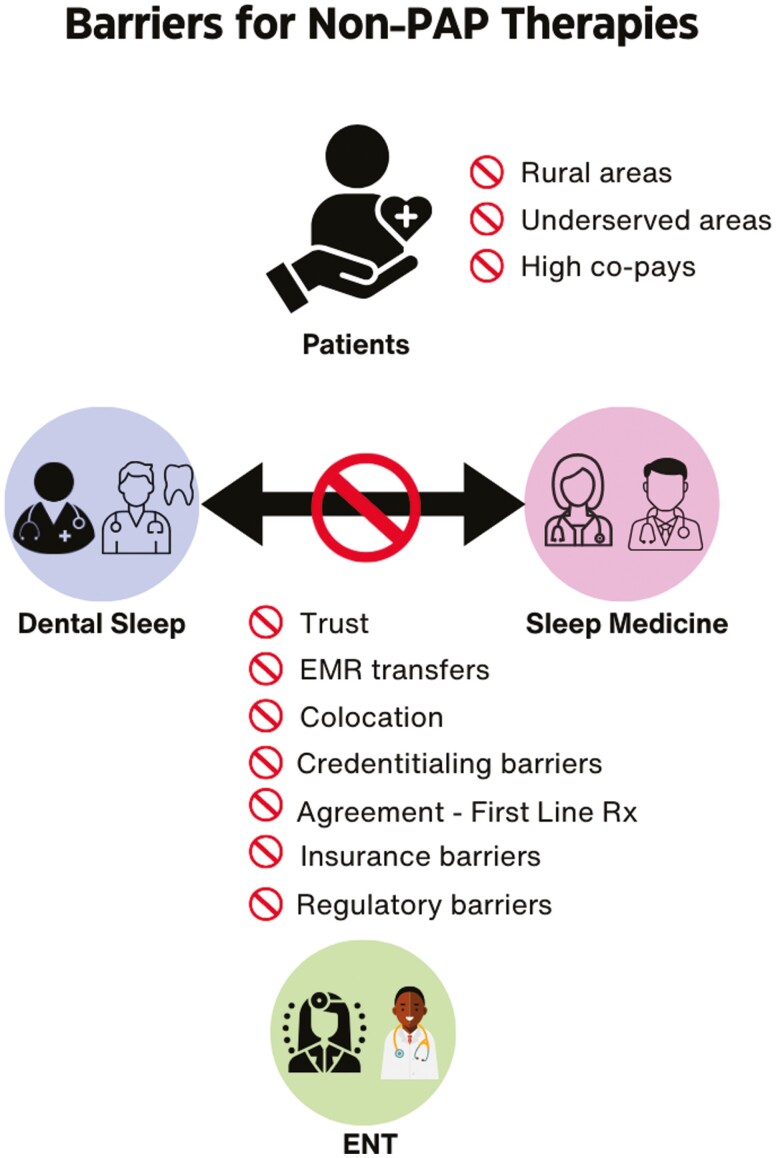
Barriers to multidisciplinary coordinated care for non-positive airway pressure (non-PAP) therapies are shown in the figure. Non-PAP therapy is generally less available to patients from rural and underserved areas within urban locations and encounters higher out-of-pocket expenses (co-pays). Between the various providers in sleep medicine, dental sleep medicine, and otolaryngology (ear nose throat [ENT]) specialties there is a general lack of trust, absence of common electronic medical record (EMR) platforms, inability to co-locate due to regulatory or other barriers. Moreover, credentialing barriers of dental sleep providers for being able to order sleep study tests and insurance company policies that prevent coverage of dental devices and PAP devices within five years of each other pose additional barriers. Moreover, there are philosophical barriers across these specialty providers with regard to whether oral appliance therapy or hypoglossal nerve stimulation therapy can be considered as first-line treatment.

Clearly, a multidisciplinary care model that includes sleep medicine providers, dentists, surgeons, DME, respiratory therapists, clinical psychologists, nursing, and sleep technicians is needed for us to bring the best care to our patients with OSA. We will identify and examine the barriers and facilitators for implementing such a patient-centered model of care delivery (Table 2 and Figure 1). The various areas tackled here are not meant to be comprehensive but aimed at addressing major thematic areas that emerge immediately. In many ways, a critical evaluation in this space requires a conceptual framework from the field of implementation science for a deliberate and methodical evaluation aimed at addressing the creation of a multidisciplinary approach for treating patients with OSA [81, 82]. We propose the RE-AIM framework that addresses the following key domains—reach, effectiveness, adoption, implementation, and maintenance [83]. Briefly, the “reach” signifies how many patients (end-users) could benefit from this program and how representative of the population are the patients who would benefit from such a multidisciplinary approach. The “effectiveness” of the multidisciplinary care model would relate to whether the initiative would achieve its key outcomes, which in this case would be to provide highly patient-centered care. The “adoption” refers to the settings (practices or healthcare organizations) that can use the multidisciplinary care model. The “implementation” arena pertains to how the multidisciplinary model will be implemented through adaptation, operationalization, and delivered with the original intent intact. Lastly, the “maintenance” aspect would pertain to whether such a multidisciplinary care program is sustainable and stable in the long term. Many of the items listed in Table 2 are self-explanatory and will not be repeated in the text here, but it would suffice to say that the various complexities that range from regulatory, reimbursement, personnel, resources, and a multitude of stakeholders’ opinions warrant deliberate and thoughtful implementation [84]. An important barrier to greater multidisciplinary model of care is the access to, and availability of, the sleep medicine provider who will need to serve an important role in shepherding patients through a complex workflow. Although models of care with primary care providers serving a central role have been suggested to be non-inferior to sleep specialists, the assessments and decision-making made by individuals without training in sleep medicine may be infeasible considering the evolving complexities of OSA treatments [22, 85]. An alternative may be to establish partnerships with primary care providers, but the nascent nature of clinical decision support systems in the sleep field combined with literature that suggests better patient outcomes of patients receiving care from specialists versus non-specialists raises concerns for sleep medicine provider’s availability is a remaining barrier [86–88].

**Table 2.** Barriers and Facilitators for Adoption of Non-PAP Therapies for OSA

Barriers^*^	Facilitators
Dental practice	
Fragmentation of care due to regulatory agencies that prohibit colocation and employment (Effectiveness and adoption)	Stronger collaboration through credentialing but not employment.
Different electronic medical records systems (implementation)	Access to electronic medical records systems
Reimbursement denials for OAT if there was a recent approval for PAP device < 5 years ago. (implementation)	Prompt return of PAP device within 90 days [79]
Out-of-pocket costs pose a barrier for patients with dental insurance. (reach)	Dental practice to bill medical insurance for OAT
Many dental practices are foregoing Medicare patients due to high regulatory cost and low reimbursements. (Adoption and reach)	Medicare to reduce regulatory cost on dental practice and consider value-based bundled care models.
Lack of trust as to whether eligible patients with OSA are being referred to OAT. (implementation)	Develop and operationalize a “verbal” inter-disciplinary contract that would require a workflow that would lead to prompt referrals from sleep center to dental sleep practice.
Lack of credentialing and privileging to assess patient outcomes and order sleep studies. (implementation)	Collaborate as part of a multidisciplinary practice with sleep medicine providers.
Sleep medicine practice	
Lack of trust as to whether there will be routine sleep study testing after titration of OAT. (implementation)	Develop and operationalize a consensus approach to co-managing patients with OSA that would require the multidisciplinary team to evaluate the treatment efficacy of OAT by performing a home sleep study [80].
Disagreement on whether OAT can be first-line therapy. (adoption)	Develop and operationalize a consensus approach to co-manage patients with OSA. AASM and AADSM to work more strongly together to not only develop, but assist with implementation aspects of operationalizing a multidisciplinary approach to treat patients with OSA.
Reimbursement denials for PAP device if there was a recent approval for OAT < 5 years ago. (implementation)	Advocacy for changes in health policy.
Reduced number of, and access to, sleep medicine providers.	Partnership with primary care physicians and embracing clinical decision support systems
Dental and sleep medicine practice	
Long-term sustainability (maintenance)	Recognition from patient communities of providing highly patient-centered care and financial viability
Access to multidisciplinary care in under-resourced areas including rural settings—health disparities (adoption and reach)	Mobile health units if financially feasible.
Program development costs (adoption)	Investments into multidisciplinary care by healthcare systems.
ENT practice	
Integration of care with sleep physician and laboratory	Common electronic medical records and institution
Training and certification of sleep physicians and sleep technicians to manage and test outcomes of implanted HNS devices	Workshops conducted by professional societies and industry Certification of sleep physicians
Personnel expenses related to care coordination	Practice agreements between ENT surgeons and sleep physicians

^*^Domains from the RE-AIM framework have been used to categorize the domain on which these barriers will have the most influence. Some of these barriers may affect other domains within the RE-AIM framework as well. PAP = positive airway pressure therapy; OAT, oral appliance therapy; AASM, American Academy of Sleep Medicine; AADSM, American Association of Dental Sleep Medicine.

An international perspective on each of these barriers and facilitators lends further insight. For example, in Canada, the workflow of attempting PAP therapy before OAT or vice versa is not met with barriers due to arbitrary regulatory policies that prohibit the second therapy for a period of 1–5 years. Such US healthcare policies are not at all patient-centered and are likely aimed at achieving cost control and limiting healthcare utilization. A detailed outline of a suggested model and workflow at a single center with a multidisciplinary care model has been proposed by Sharma et al [89] and provides a roadmap for others to emulate. While Table 2 addresses barriers and facilitators for sleep medicine and dental sleep medicine care, similar framework and evaluation approaches can be developed for planning and evaluating the surgical aspects of a multidisciplinary program that includes HNS and bariatric surgery.

An important yet unanswered question is whether patients receiving a “rescue” therapy for OSA (regardless of category) after a failed attempt at receiving a different initial therapy are disadvantaged and whether we should develop a prediction tool for selecting the right treatment for the right patient at the right time—precision sleep medicine. Phenotyping patients both from the standpoint of symptomatology (sleepiness, etc.), physiological parameters (Pcrit, loop gain, muscle responsiveness, arousal threshold, etc.), and other factors (anthropometric features such as BMI, OSA severity, upper airway anatomy, and response to drug-induced sleep endoscopy [DISE]), would lend itself to precision medicine approaches in the sleep field.

There are precision medicine approaches that are already being undertaken in selecting patients with OSA for non-PAP treatment alternatives. For example, DISE is predictive but not perfect for selecting patients for HNS [62]. Similarly, a remotely controlled mandibular positioner (MATRx) test has been demonstrated to be associated closely with the treatment outcome in patients with OSA undergoing a laboratory-based sleep study [90] Unfortunately, this device is no longer commercially available. Other approaches such as the OAT “boil and bite” can also be attempted but there are concerns regarding the predictive ability and reproducibility of such approaches [91]. A randomized clinical trial designed to compare ready-made versus custom-made mandibular repositioning devices found that the custom appliances were superior in reducing both the oxygen desaturation index and the apnea–hypopnea index (AHI) and resulted in complete response to therapy more often than the ready-made appliances [91]. Patient preference was also heavily weighted on the side of the custom-made appliance.

Myofascial pain can develop with OAT when the patient’s mandible is repositioned too quickly. To avoid such a situation, it is important to remain within the adaptive capacity of the musculature involved with mandibular repositioning. This capacity is elastic, and in some cases, the patient would not be able to immediately comfortably maintain their optimum position, but would, given the passage of sufficient time, and such time varies significantly between patients. This issue makes the immediate determination of optimum mandibular position difficult for patients who require more time to adapt to their optimum position. Another approach that uses a combination of tongue size and sex to select phenotypical features that achieve treatment outcomes superior to the conventional fitting of custom oral appliances is available but warrants careful implementation and evaluation in new settings (ApneaGuard). The ApneaGuard trial device (Advanced Brain Monitoring, Carlsbad CA) was developed to predict both efficacy and optimum mandibular position, including the degree of vertical opening [92]. A better understanding of other treatment response predictors such as the identification of phenotypes that confer greater responsiveness to OAT or HNS needs to be considered. Studies have shown that responders to OAT are more likely to have a lower BMI, smaller neck circumference, lower apnea–hypopnea index, greater retraction of the maxilla and mandible, a narrower airway and a shorter soft palate, and subdued ventilatory responses than non-responders [93, 94]. Similarly, studies of HNS therapy have clearly suggested that individuals with the absence of complete collapse of soft palate as seen during a DISE, no other anatomical obstructions (e.g. enlarged tonsils), BMI < 35 Kg/m2, and low levels of central or mixed events of OSA are more likely to respond to treatment [62, 95].

The cost-effectiveness of OAT and HNS when compared to PAP therapy has been undertaken but requires further study [96]. For each of the non-PAP approaches (OAT and HNS) one needs to factor in the costs of the diagnostic test, device, and repeat testing in relation to effectiveness of the treatment in order to generate the proof that the device is cost-effective.

## Limitations

We have not addressed certain special case scenarios such as how to handle complex patients with underlying cardiopulmonary problems (e.g. hypoventilation phenotype with less upper airway resistance problems and underlying heart failure or severe chronic obstructive pulmonary disease). The purpose of this white paper is to bring to the forefront the complex nature and need for a multidisciplinary approach to managing patients with OSA and future research and endeavors needed to address such special circumstances. Moreover, we did not address other non-PAP therapies such as external tongue stimulators (ExciteOSA), upper airway surgeries, medical weight loss, bariatric surgery, recent pharmacological agents for OSA, and positional devices considering that they are not as commonly used as OAT and HNS therapies and/or that more robust clinical trial data is still needed [97]. Considering that our a priori stated aim of the focus group was to assess HNS and OAT therapy we had not assembled dieticians, exercise physiologists or obesity specialists and addressing such non-PAP treatment approaches at this late stage is not feasible without conducting a new focus group.

## Future Directions

Implementation trials for the early evaluation and subsequent operationalization of a multidisciplinary care model that includes non-PAP treatment alternatives for OSA need to be undertaken with careful assessment of both process and patient (client) outcomes (Table 3). Moreover, there is a clear need for comparative effectiveness research with head-to-head trials of various OSA therapies that are adequately powered to address the relative benefits and appropriate candidates for such therapies [98]. Such trials could inform future precision medicine approaches for better patient-centered care. Such research will also need to inform changes in healthcare policies that currently continue to fragment care, withhold care, and perpetuate health disparities [52, 99, 100]. Other important knowledge gaps that lend themselves to study across the clinical-translational spectrum of efficacy, comparative effectiveness, health policy, and health services research are identified in Table 3. The desired changes in healthcare policies; however, will not be possible through research alone as such change needs strong collaboration between professional medical societies that encompass sleep medicine, dental sleep medicine, otolaryngology, bariatric surgery, and sleep research. Collaborative engagement of these professional medical societies through the Association of Professional Sleep Societies and programming at the annual meeting that addresses this important issue in the sleep field is desperately needed to advance scientific understanding, and patient-centered outcomes, that will enable us to serve our patients well.

**Table 3.** Future Research Opportunities

Efficacy studies	
	Better phenotyping aimed at identifying treatment responders to OAT and HNS
Comparative effectiveness research	
	Comparative effectiveness research with head-to-head trials of various OSA therapies that are adequately powered to address the relative benefits and appropriate candidates for such therapies
	Adaptive precision-medicine clinical trials based upon prediction of treatment responders
Implementation science	
	Hybrid Type 2 or Type 3 implementation trials that assess comparative-effectiveness of various models of care delivery (e.g. multidisciplinary versus conventional discipline-based approaches [i.e. PAP therapy or dental or HNS approaches alone])
	Identification of barriers and facilitators through qualitative and mixed methods approaches
Health-policy research	
	Observational or intervention-based approaches aimed at improving access to care for Non-PAP therapy modalities such as OAT or HNS therapies
	Identifying or addressing (through interventions) the fragmentation of care delivery (e.g. disjointed care and delivery systems across sleep, dental, ENT specialists, durable medical equipment companies), withholding of care (e.g. denial of OAT when PAP therapy fails and 90-day rule for denial of PAP benefits) all of which perpetuate sleep health disparities.
Patient-centered outcomes	
	Studying patient preference for OSA treatment, patient satisfaction ratings, and treatment adherence in addition to traditional physiological end-points (e.g. apnea–hypopnea index, patient-reported outcomes, and cardiovascular morbidities).
Health services research	
	Understanding practice trends and healthcare utilization.
	Studying out-of-pocket expenses and insurance coverage for non-PAP therapies (OAT and HS) in relation to PAP therapy.
	Cost-effectiveness studies that compare OAT, HNS, and PAP therapy

OAT, oral appliance therapy; HNS, hypoglossal nerve stimulation; OSA, obstructive sleep apnea; ENT, ear nose throat specialists;.

## Data Availability

There are no original data for this review article to be shared.

## Appendix: Probes used for the focus group

1. Definition of PAP Treatment Failure?
  a. What is the threshold of treatment failure and when to consider alternative therapy?
  b. Should we use the AHI at all? or other criteria like oximetry-based metrics and self-reported outcomes?
  c. Does treatment failure with one therapy adversely affect future treatment adoption by the patient or treatment adherence?
2. First Line Treatment?
  a. Is CPAP the gold-standard / first-line treatment? (AHRQ report, SAVE study, and meta-analyses in the Journal of American Medical Association)
  b. Should non-PAP alternatives be offered as first-line therapies?
  c. Is there reluctance on the part of sleep physicians for offering non-PAP alternatives? If so, what are the barriers and facilitators?
  d. How does one select patients for such a “first-line” approach? [PATIENT SELECTION]
  e. How does patient-preference factor into this approach?
3. Combining Therapies
  a. Is “combining therapies” utilized as often as it could be to optimize patient outcomes? If not, why not? Is it warranted? What can be done to change this?
4. Goals of therapy
  a. Optimal outcome measures of alternative therapy in clinical trials?
  b. Which intermediary outcome measures are most important to reduce morbidity: oximetry, AHI, snoring, work of breathing, sleep fragmentation, biological markers, other?
  c. Which signal in oximetry measures is most important: nadir O2 saturation, ODI, mean O2 saturation, saturation index time below 88%?
  d. What is the threshold for oxidative stress in general, such as 2% of TST below 88%?
5. Multidisciplinary care model
  a. How can sleep centers be multidisciplinary (have a closed loop referral system) to include dentists prescribing dental device and ear nose throat surgeons implanting HGNS?
  b. How to foster a collaborative environment?
  c. Timely second line treatments can be instituted. So that ALL patients are treated. The 5-year rule after PAP failure for MAD coverage!
  d. How can treatment effectiveness can be comprehensively assessed.
  e. Dental device DME and related regulatory issues
  f. Laboratory-based titration (Dental device and HGNS)
  g. Call triage between team members
6. Special cases
  a. How to handle hypoventilation phenotype with less upper airway resistance problems?
  b. Edentulous individual?
  c. Temporo-mandibular joint problems
  d. Co-morbid medical conditions (COPD, etc.)?
